# Pharmacokinetic Comparison of Scutellarin and Paeoniflorin in Sham-Operated and Middle Cerebral Artery Occlusion Ischemia and Reperfusion Injury Rats after Intravenous Administration of Xin-Shao Formula

**DOI:** 10.3390/molecules21091191

**Published:** 2016-09-07

**Authors:** Yueting Li, Yuan Lu, Jianchun Hu, Zipeng Gong, Wu Yang, Aimin Wang, Jiang Zheng, Ting Liu, Tingting Chen, Jie Hu, Ling Mi, Yongjun Li, Yanyu Lan, Yonglin Wang

**Affiliations:** 1 Key Laboratory of Pharmaceutics of Guizhou Province, Guizhou Medical University, No.9, Beijing Road, Yunyan District, Guiyang 550004, China; nhwslyt@163.com (Y.L.), 18798090340@163.com (Y.L.), jianchun89@163.com (J.H.), gzp4012607@126.com (Z.G.), 18798090340@163.com (W.Y.), zhengneu@yahoo.com (J.Z.), t-liu@163.com (T.L.), 18798846132@163.com (T.C.), hujie51619@sina.cn (J.H.), 18798860432@163.com (L.M.); 2 National Engineering Research Center of Miao’s Medicines, Guiyang 550004, China; 3 School of Pharmacy, Guizhou Medical University, No. 9, Beijing Road, Yunyan District, Guiyang 550004, China; 4 Engineering Research Center for the Development and Applications of Ethnic Medicines and Traditional Chinese Medicine (TCM), Ministry of Education, Guizhou Medical University, No. 9, Beijing Road, Yunyan District, Guiyang 550004, China; gywam100@163.com (A.W.); liyongjun026@126.com (Y.L.); yanyu626@126.com (Y.L.)

**Keywords:** Xin-Shao formula, scutellarin, paeoniflorin, MCAO, pharmacokinetics, UPLC-MS/MS

## Abstract

Xin-Shao formula is a folk remedy widely used in China to prevent and cure stroke. Cerebral ischemic reperfusion (I/R) injury often takes place during the treatment of stroke. Information about the pharmacokinetic behavior of the remedy under cerebral I/R injury conditions is lacking. The present study aimed to compare the pharmacokinetic properties of scutellarin and paeoniflorin, two major bioactive components of Xin-Shao formula, under physiological state in cerebral I/R injury rats. Neurobehavioral dysfunction was evaluated and cerebral infarcted volume was measured in middle cerebral artery occlusion I/R injury (MCAO) rats. Plasma samples were collected at various time points after a single dose (intravenous, i.v.) of Xin-Shao formula. The levels of plasma scutellarin and paeoniflorin at the designed time points were determined by a UPLC-MS/MS method, and drug concentration versus time plots were constructed to estimate pharmacokinetic parameters. Increase in terminal elimination half-life (t_1/2z_) and mean residence time (MRT_(0–t)_) of scutellarin as well as elevation in area under the plasma drug concentration-time curve from 0 h to the terminal time point (AUC_(0–t)_) and maximum plasma drug concentration (C_max_) of paeoniflorin, along with decreased clearance of paeoniflorin and scutellarin as well as reduced apparent volume of distribution (V_z_) of paeoniflorin, were observed in MCAO rats, compared with those in sham-operated animals. The elimination of scutellarin and paeoniflorin were reduced in cerebral I/R injury reduced rats.

## 1. Introduction

Stroke, defined as a sudden neurological dysfunction in the brain caused by either ischemia (lack of blood supply to the brain) or a hemorrhage, has become one of the leading causes of severe neuronal impairment, loss of brain functions, and death [1]. Around 80% of strokes result from cerebrovascular system blockage (ischemia) [2]. Reperfusion is considered as an effective approach to improve the ischemic condition of the brain, particularly for vascular blockage, however, cerebral ischemia and reperfusion (I/R) may induce an accumulation of reactive oxygen species released from mitochondria, which possibly accelerates lipid peroxidation and the process of neuronal death [3]. The resulting oxidative stress could worsen the extent of stroke [4].

Many traditional Chinese medicines (TCMs) are reported to facilitate recovery from stroke [4]. Xin-Shao formula, consisting of *Erigeron breviscapus* (Vant.) Hand.-Mazz and *Paeonia veitchii* Lynch, is a common TCM and has been used for the prevention and treatment of acute brain infarcts and stroke [5,6,7]. Our early studies demonstrated that Xin-Shao formula remarkably inhibited the arteriovenous bypass thrombogenesis in rats [8], protected against cerebral I/R injury in rats [9], improved microcirculation disturbance resulting from blood deficiency in ischemic site of brain [9], and decreased reactive oxygen species in middle cerebral artery occlusion (MCAO) rats [9] and in cultured PC12 cells [10].

**Figure 1:**
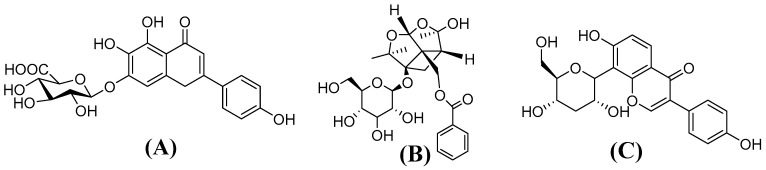
The structures of scutellarin (**A**); paeoniflorin (**B**); and puerarin (**C**, IS).

A pharmacokinetic study of Xin-Shao formula has been performed in normal rats in our laboratory [11]. However, the pharmacokinetic behaviors of the herbal medicine are not necessarily the same under pathophysiological conditions, specifically in cerebral I/R injury. Cerebral I/R injury is commonly recognized as a major factor which could be an obstacle to effective cure of stroke. Cerebral I/R injury was reported to alter pharmacokinetic behaviors of some herbal medicines [2,12,13]. The primary objective of this study was to compare pharmacokinetic properties of Xin-Shao formula in cerebral I/R injury model rats and sham-operated rats. Scutellarin (Figure 1A) and paeoniflorin (Figure 1B), two major components known to protect against cerebral I/R injury [14,15,16,17,18,19,20], were monitored for the pharmacokinetic study. We hoped the study would provide useful information to guide the effective use of Xin-Shao formula in the clinic.

## 2. Results

### 2.1. Neurobehavioral Abnormality and Cerebral Infarcted Volume

**Figure 2:**
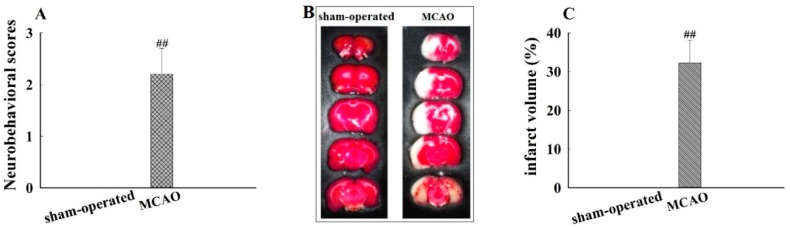
Neurobehavioral disability and infarct regions caused by middle cerebral artery occlusion I/R injury (MCAO), evaluated by neurobehavioral scores and 2,3,5-triphenyltetrazolium chloride (TTC) staining. (**A**) Neurobehavioral scores; (**B**) TTC staining of brain; and (**C**) infarct volume (%). Rats exposed to 1 h ischemia followed by 24 h reperfusion to establish MCAO model. Neurobehavioral disability of rats was assessed (*n* = 12), and brain tissues were collected for TTC staining (*n* = *6*). Data are mean ± standard deviation (SD), ##: *p* < 0.01 for MCAO vs. sham-operated group.

The neurological damage induced in rat model was evaluated by monitoring neurobehavioral dysfunction, using the five point scale. The degree of neurobehavioral dysfunction varied in MCAO-induced rats from one to another. Some of the MCAO-induced rats failed to fully flex the right forepaw, circled counter clockwise, or leaned to the left side. The scores were 0 vs. 2.2 ± 0.5 (*p* < 0.01) for sham-operated and MCAO groups, respectively (Figure 2A). Additionally, cerebral damage induced by I/R was observed in MCAO rats (Figure 2B). Specifically, the infarcted volume (%) of the I/R was 0 vs. 32.3 ± 5.8 (*p* < 0.01) for sham-operated and MCAO groups, respectively (Figure 2C). Taken together, these results indicated that the MCAO model was successfully established.

### 2.2. Method Validation

#### 2.2.1. Specificity

**Figure 3:**
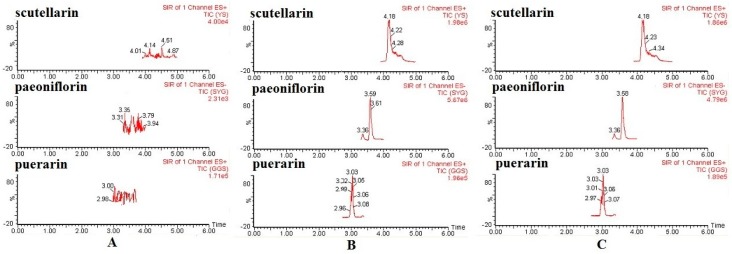
Typical selected ion recording (SIR) chromatograms obtained from analyses of blank plasma (**A**); blank plasma spiked with the scutellarin, paeoniflorin, and IS (**B**); and plasma samples from normal rats 15 min following the intravenous administration of Xin-Shao formula extracts (**C**).

No interference was observed in the retention times of scutellarin, paeoniflorin, and puerarin (IS) in all plasma samples analyzed, and the method exhibited good specificity. Typical SIR chromatograms are shown in Figure 3.

#### 2.2.2. Linearity, LLOQ, Precision, and Accuracy

The typical regression equation of calibration curves were as follows: *y* = 0.6465*x* + 0.290 (*r* = 0.9979 for scutellarin) and *y* = 0.5017*x* + 0.441 (*r* = 0.9973 for paeoniflorin) The results showed good linearity over the range of 0.02899–59.37 μg/mL and 0.02950–60.42 μg/mL for scutellarin and paeoniflorin, and their lower limit of quantification (LLOQ) values were 27 and 23 ng/mL.

The intra- and inter-day precision and accuracy data of the assays are shown in Table 1. The intra-day and inter-day relative standard deviation (RSD) were 4.1% to 8.9% for scutellarin and were 4.4% to 7.6% for paeoniflorin respectively. Meanwhile, the corresponding accuracy ranged from 94.25% to 101.3% for scutellarin and 94.07% to 101.5% for paeoniflorin, indicating that all the values were within acceptable criteria.

#### 2.2.3. Recovery and Matrix Effects

As shown in Table 2, the mean extraction recoveries of scutellarin and paeoniflorin and were all more than 84.77% at three QC concentration levels, which indicates that the recoveries of the analytes were consistent and reproducible. The mean extraction recovery of the IS was 83.1%. Moreover, the results of matrix effects (Table 2) demonstrated no significant ion enhancement or suppression observed for the two analytes.

#### 2.2.4. Stability

The stability study showed (Table 3) that both the analytes were stable under all conditions tested, including short-term storage (6 h at 24 °C), freeze-thaw cycles (from −20 °C to 20 °C) on consecutive 3 days, and long-term storage (−20 °C for 2 weeks). The RSD values of the stability rates were less than 8.8%. Therefore, the method has been proved to be applicable for routine analysis.

### 2.3. Pharmacokinetic Analysis

**Figure 4:**
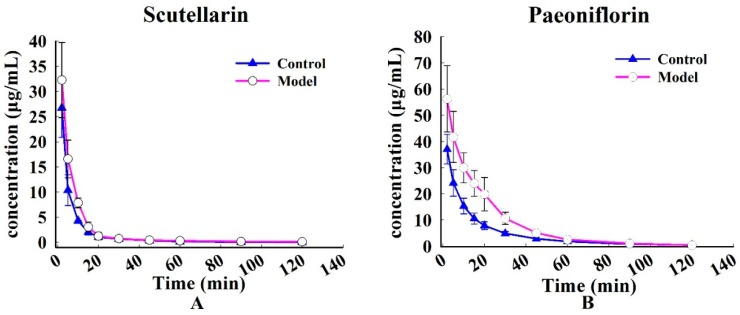
The mean plasma concentration vs. time profile of the two components following the intravenous administration of Xin-Shao formula extracts in sham-operated and MCAO rats. (**A**): scutellarin and (**B**): paeoniflorin. Each value represents mean ± SD (*n* = 6).

The profiles of time-course mean plasma concentrations of scutellarin and paeoniflorin following intravenous administration of Xin-Shao formula extracts are shown in Figure 4. The two compounds vanished rapidly in rats after injection. The main non-compartmental parameters are summarized in Table 4.

After intravenous administration of Xin-Shao formula extracts, mean residence time (MRT_(0–t)_) (0.42 ± 0.15 min) and terminal elimination half-life (t_1/2z_) (1.66 ± 0.68 min, *p* < 0.05) for scutellarin in the MCAO group were significantly prolonged relative to that of the sham-operated group (0.26 ± 0.09 min and 0.81 ± 0.48 min). Furthermore, area under the plasma drug concentration-time curve from 0 h to the terminal time point (AUC_(0–t)_) (18.35 ± 3.71 mg·min·L^−1^) and maximum plasma drug concentration (C_max_) (56.28 ± 12.62 mg·L^−1^, *p* < 0.01) for paeoniflorin in the MCAO rats were significantly higher than those of the sham-operated rats (10.35 ± 2.41 mg·min·L^−1^ and 36.40 ± 5.71 mg·L^−1^); on the contrary, decrease in apparent volume of distribution (V_z_) of paeoniflorin was observed in MCAO rats (0.21 ± 0.08 L·kg^−1^), compared with those in sham-operated animals (0.43 ± 0.07 L·kg^−1^, *p* < 0.01). However, the disappearance of scutellarin and paeoniflorin plasma was slower in MCAO rats than in sham-operated rats. Lower values of clearance (CL_z_) (0.97 ± 0.26 vs. 1.57 ± 0.60 L·min·kg^−1^, *p* < 0.05) for scutellarin and (0.45 ± 0.11 vs. 0.78 ± 0.16 L·min·kg^−1^, *p* < 0.01) for paeoniflorin were observed in MCAO rats than those in sham-operated rats.

## 3. Discussion

Cerebral I/R injury was reported to induce liver injury and hepatic dysfunction which results from an excessive accumulation of reactive oxygen species [21], activation of apoptosis [22], energy deficiency [22], and inflammatory reaction [23]. The resulting hepatic energy deficiency and apoptosis even necrosis may cause alternation in functions of drug-metabolizing enzymes and transporters [23], which possibly affect drug biotransformation and disposition.

The major hepatic metabolic pathways for scutellarin include hydrolysis and glucuronic acid conjugation [24]. The hydrolysis of scutellarin by β-glucuronidase offers scutellarein (aglycone) and glucuronic acid. The later pathway produces the corresponding glucuronide [24], and UDP-glucuronosyltransferases (UGTs) are the enzymes responsible for the formation of glucuronides. Glucuronidation could be an important factor commanding the elimination of drugs [25]. The conjugation process of scutellarin was mainly mediated by UGT1A9, 1A1 and 1A8 [26]. Glucuronidation is a conjugative clearance system with a large capacity and plays a dominant role in the elimination of scutellarin [24]. The expression of UGT1A9 and 1A1 was reported to be down-regulated in response to pathophysiological conditions such as inflammatory reaction [27] which often takes place during cerebral I/R injury [28]. The present study showed that the disappearance of scutellarin in plasma was slower in MCAO rats than in sham-operated rats. The higher values of t_1/2z_ and MRT_(0–t)_ found in MCAO rats than sham-operated rats possibly attributed to the reduction in the elimination of scutellarin, and such reduction might arise from the decreased expression of UGT1A9 and 1A1 reported.

Expression of cytochromes P450s 3A [29], 2E1 [30], and 2B [31] were reportedly down-regulated in MCAO rats. As a result, the metabolism of those drugs mainly catalyzed by P450s 3A, 2E1, and 2B may be slowed down in MCAO rats. The present study clearly demonstrated that the disappearance of paeoniflorin in plasma was slower in MCAO rats than in sham-operated rats, which indicates that alteration induced by cerebral I/R injury of Xin-Shao formula extracts in pharmacokinetic behavior was evident. Paeoniflorin is mainly metabolized by a number of P450 enzymes, such as P450s 3A4, 2C9, and 2C8, and P450 3A4 was reported to be the primary enzyme responsible for the metabolism of paeoniflorin in liver [32]. The observed slow disappearance of plasma paeoniflorin in MCAO rats likely resulted from slowed metabolism of paeoniflorin mediated by P450 3A2, which reduces non-renal clearance of paeoniflorin. When drugs were administered intravenously, metabolism occurring in liver is a major factor dictating the fate of drugs [33]. Accordingly, the observed higher values of AUC_(0–t)_ and C_max_ in MCAO rats than sham-operated rats as well as decreased V_z_ in MCAO rats possibly resulted from decreased elimination of paeoniflorin.

## 4. Materials and Methods

### 4.1. Chemicals and Reagents

*E. breviscapus* herba material was collected from Honghe, Yunnan Province, China in November, 2014 and *P. veitchii* radix rubra was harvested from Deyang County, Sichuan Province in October, 2014. The herbal medicines were authenticated by Qingde Long of Guizhou Medical University and voucher specimens with accession numbers 20141116 and 20141028 were deposited at the Guizhou Medical University. The herbal medicines was subsequently sun-dried and ground. Scutellarin (purity 98%), paeoniflorin (purity 99%), and puerarin (purity 98%) were purchased from the National Institute for Food and Drug Control (Beijing, China). 2,3,5-Triphenyltetrazolium chloride (TTC) was from Sigma-Aldrich (Saint Louis, MO, USA). Methanol, acetonitrile, and formic acid with HPLC-grade were obtained from Merck KGaA Co. (Darmstadt, Germany). All other chemicals and reagents were of chromatographic grade from Tianjin Kemiou Chemical Reagent Corp. (Tianjin, China). Deionized water was obtained using an EPED super-purification system (EPED, Nanjing, China).

### 4.2. Preparation of Xin-Shao formula Extracts

*E. breviscapus* herba was thoroughly soaked in water for 30 min. The soaked herb was decocted in water (1:10, *w*/*v*) for 0.5 h, followed by filtration. The filtrates were collected and the remaining was re-decocted in water (1:10, *w*/*v*) for 0.5 h. The process was repeated twice. The three filtrates were pooled and condensed under reduced pressure to concentrates with a relative density of 1.09–1.11 (50 °C). The resulting concentrates were mixed with ethanol (final concentration 55%) and were allowed to stand at room temperature for 12 h to precipitate protein and polysacchrides. The precipitates were removed by pump filtration. The ethanol in the filtrates was removed under reduced pressure and the residue was subsequently concentrated until the mixture showed a relative density of 1.10–1.12 (50 °C). The resulting concentrates were acidified with hydrochloric acid to pH 2, and the solution was kept at 50 °C for 6 h. The resulting precipites were harvested, washed with water until the aqueous phase showed pH 3–4, and lyophilized to dryness and stored in a desiccator.

*P. veitchii* radix rubra was thoroughly soaked in water for 30 min. The soaked radix rubra was decocted in water (1:8, *w*/*v*) for 1 h, followed by filtration. The filtrates were collected and the remaining was re-decocted in water (1:8, *w*/*v*) for 1 h. The process was repeated twice. The three filtrates were pooled and condensed under reduced pressure to concentrates with a relative density of 1.06–1.08 (50 °C). The resulting concentrates were mixed with ethanol (final concentration 60%) and were allowed to stand at room temperature for 12 h to precipitate protein and polysacchrides. The precipites were removed by pump filtration. The ethanol in the filtrates was evaporated under reduced presure and the residue was subsequently concentrated until the mixture showed a relative density of 1.18–1.20 (50 °C). Then, the filtrates were leach-extracted four times with *n*-butanol (BuOH), and the alcoholic layers were collected and pooled, followed by washing with water three times. The organic solvent was removed under reduced pressure. The resulting residue was dissolved in 45% ethanol and loaded onto a polyamide column. The resultant column was eluted with 45% ethanol, and the eluates were collected. The ethanol was subsequently removed by evaporation, and the resulting precipitates were dried under reduced pressure. The resulting dry extracts obtained from *E. breviscapus* and *P. veitchii* were mixed at a ratio of 2:3 (*w*/*w*). The mixture was reconstituted in saline, autoclaved, filtered, and lyophilized to dryness before use.

### 4.3. Establishment and Evaluation of MCAO Rat Model

Male Sprague-Dawley rats, weighing 280–300 g, were purchased from the Charles River Laboratories (Beijing, China), and allowed unlimited access to food and water while maintained in an air-conditioned animal center at a temperature of 22 ± 2 °C, relative humidity of 60% ± 10%, and on a natural light-dark cycle. The animals were fasted with access only to water for 12 h prior to the experiments. The animal welfare and experimental procedures adhered to the Guide for the Care and Use of Laboratory Animals [34]. Furthermore, the experimental animal protocol was approved by the Animal Ethics Committee of Guiyang Medical University.

#### 4.3.1. Establishment of MCAO Model

Animals were randomly divided into two groups consisting of the MCAO and sham-operated groups. The MCAO rat model was used to mimic an ischemic stroke and this was induced, according to a published procedure [35] with some modification. Briefly, animals were anesthetized with 10% chloral hydrate (350 mg/kg, intraperitoneal, i.p.), and then the left common carotid artery (CCA), external carotid artery (ECA), and internal carotid artery (ICA) of the rats were carefully separated from tissues and adjacent nerves by a cervical incision. At the proximal part of CCA and ECA, permanent knots were tied to prevent the backflow of blood. Then, ICA was temporarily clamped with microsurgical clips. The tip of a 60 mm long polylysine coated nylon monofilament was rounded by heating near a glowing ember (final tip diameter, 0.38 ± 0.02 mm, Beijing Sunbio Biotech Co., Ltd., Beijing, China) and was inserted into the arteriotomy hole made on the distal part of CCA. Then the monofilament was gently impelled close to 18–20 mm from CCA to block the blood supply to MCA and achieve cerebral ischemia. Sequentially, the rats were cannulated with a polyethylene catheter (0.50 and 1.00 mm for inside diameter and outside diameter, Portex Ltd., Hythe, UK) in the right jugular vein for pharmacokinetic analysis. After induction of cerebral ischemia for 1 h, a 24-h reperfusion was performed by gently pulling out the filament. The sham-operated rats experienced the same surgical operations except for no nylon monofilament inserted. Throughout the entire surgical procedure, the environmental temperature was maintained at 37 ± 1 °C.

#### 4.3.2. Evaluation of Neurobehavioral Dysfunction

Behavioral changes were assessed immediately after the MCAO rats received a 24 h reperfusion. Neurobehavioral dysfunction was double-blindly evaluated in sham-operated and MCAO rats (*n* = 12 per group), using Longa’s five-point scale [35] as follows: 0, normal (no neurobehavioral dysfunction); 1, slight (failure to fully flex the right forepaw); 2, moderate (circling counter-clockwise); 3, severe (leaning to the left side); and 4, very serious (no autonomous activity and rat is unconscious).

#### 4.3.3. Measurement of Cerebral Infarction Volume

After evaluation of neurobehavioral dysfunction, six animals in each group were randomly selected to measure the cerebral infarction volume. The cerebra were immediately frozen at −20 °C for 15 min and were sliced into five 2-mm sections. The tissue sections were incubated in a 1% solution of TTC at 37 °C for 20 min and then fixed in 4% paraformaldehyde overnight protected from light by following the instructions of the manufacturer. The viable tissue was stained a deep red while the infarct remains unstained [36]. The TTC-stained slices were photographed, and the infarction volume was measured using an image analysis software (Image-Pro Plus 5.1, Media Cybernetics, Silver Spring, MD, USA). Infarcted volume (%) was calculated according to methods described by Ding et al. [37].

### 4.4. Apparatus and Analytical Conditions

An Acquity UPLC^™^ system equipped with a binary pump, degasser, autosampler, and temperature-controlled column compartment and a TQD quantum triple-quadrupole mass spectrometer equipped with an electrospray ionization (ESI) source (Waters Corp., Manchester, UK) were used to analyze the samples. The two analytes and the internal standard (IS, puerarin) were chromatographically separated on a Waters Acquity BEH C_18_ column (2.1 × 50 mm, 1.7 μm, Waters, Wexford, Ireland). The analysis was completed with a gradient elution with solvent A (acetonitrile containing 0.1% formic acid) and solvent B (water containing 0.1% formic acid) within 8.5 min at a flow rate of 0.35 mL/min. The gradient elution schedule was as follows: 0–1 min (2%–5% A), 1–3 min (5%–15% A), 3–4 min (15%–20% A), 4–5 min (20%–35% A), 5–6.8 min (35%–90% A), 6.8–7.8 min (90%–35% A), and 7.8–8.5 min (35%–2% A). The column and autosampler tray temperatures were remained at 45 and 4 °C, respectively.

For MS analysis, the ESI source was used, and the acquisition parameters were as follows: nebulizing and drying gas, nitrogen (N_2_); source and desolvation temperature, 120 and 400 °C; desolvation gas flow, 800 L/h. Selected ion recording (SIR) mode was chosen for the quantification of the analytes, and specific mass spectrometric parameters are listed in Table 5. Micromass Masslynx version 4.1 (Waters Corp., Milford, MA, USA) was used to carried out the data acquisition and processing.

### 4.5. Preparation of Calibration Standards and Quality Control (QC) Samples

A standard stock solution containing scutellarin and paeoniflorin was prepared in methanol to obtain final concentrations of 118.8 μg/mL for scutellarin and 120.8 μg/mL for paeoniflorin The IS stock solution was diluted with methanol to a final concentration of 1.00 μg/mL.

Two series of working solutions were prepared by diluting the standard stock solution with methanol. The working solutions (100 μL) were individually transfered to Eppendoff vials and evaporate to dryness under a stream of nitrogen at 37 °C. The residue was reconstituted in blank rat plasma (100 μL) to obtain calibration standard solutions with final concentrations in a range of 0.02899–59.37 μg/mL for scutellarin and 0.02950–60.42 μg/mL for paeoniflorin. The QC samples were prepared at three concentrations of 0.1160, 0.9277, and 14.84 μg/mL for scutellarin and of 0.1180, 0.9440, and 15.11 μg/mL for paeoniflorin in the same way as that for the preparation of the standard calibration samples.

### 4.6. Method Validation

#### 4.6.1. Specificity

Specificity study was performed with three test samples, including blank plasma (obtained from five rats), blank plasma spiked with the analytes and IS, and plasma samples from rats 15 min following an intravenous administration of Xin-Shao formula extracts at a dose equivalent to a crude herb dose of 1.25 g/kg.

#### 4.6.2. Linearity, LLOQ, Precision, and Accuracy

The calibration curves for scutellarin and paeoniflorin were constructed by plotting the peak area ratios (*y*) of each analyte to the IS against the plasma concentrations, and the corresponding nominal concentration (*x*) by weighted (*1/x^2^*) least-squares linear regression. The LLOQ was determined by identifying the lowest concentration of the analytes which exhibited a signal-to-noise ratio of 10:1 in UPLC-MS/MS analysis. The precision and accuracy were evaluated by five replicate analyses of the QC samples at low, medium, and high concentrations on the same day and on three consecutive days.

#### 4.6.3. Recovery and Matrix Effect

Mean extraction recovery of scutellarin and paeoniflorin was determined at three QC levels with five replicates and calculated by comparing the peak areas of the analytes from extracted samples with those from unextracted samples. For evaluation of matrix effect, the peak areas of scutellarin and paeoniflorin spiked into blank plasma were compared with those added in methanol at the QC levels with five replicates.

#### 4.6.4. Stability

The stability was evaluated by analyzing the QC samples at low, medium and high concentrations (*n* = 5) exposed to various conditions: 6 h at 24 °C, after three freeze-thaw cycles (−20 °C to 20 °C) on consecutive days, and after storage at −20 °C for 2 weeks.

### 4.7. Pharmacokinetic Study

#### 4.7.1. Drug Administration and Plasma Sample Collection

The remaining rats in each group (*n* = 6 per group), whose behavioral changes had been assessed, were given the extracts at a dose equivalent to 1.25 g crude herb per kg of rat body weight (5.12 mg scutellarin and 7.90 mg paeoniflorin per kg of body weight), which was five times equivalence of a daily dose for an adult. Blood samples (280 μL) were harvested in heparinized centrifuge tubes at designed time points (0, 2, 5, 10, 15, 20, 30, 45, 60, 90, 120, 240, 360, and 480 min) and centrifuged at 3300× *g* for 3 min. The resulting plasma was stored at −80 °C until analyzed. After each blood sample collection, 100 μL of saline containing 20 units/mL of heparin was immediately injected into the catheter to prevent coagulation.

#### 4.7.2. Plasma Sample Preparation

The plasma samples (100 μL) were mixed with 0.08 % formic acid (50 μL) and IS solution (puerarin, 15 μL, 1.0 μg/mL) and vortexed for 30 s. The resulting mixture was extracted with 360 μL of methanol by vortexing for 3 min. After centrifugation at 13,000× *g* for 10 min at 4 °C, the supernatants were concentrated to dryness with a stream of nitrogen at 30 °C. The residue was reconstituted with 150 μL of 50% acetonitrile and centrifuged at 20,000× *g* for 10 min at 4 °C. The supernatants (3 μL) were subjected to UPLC-MS/MS for analysis.

### 4.8. Pharmacokinetic Studies and Statistical Analysis

Pharmacokinetic parameters, including the relational AUC_(0–t)_, MRT_(0–t)_, t_1/2z_, CL_z_, V_z_, and C_max_, were calculated using DAS 2.0 pharmacokinetic software package (Mathematical Pharmacology Professional Committee of China, Shanghai, China). All the reported measurements were presented as the mean ± SD. The significant difference between sham-operated and MCAO groups was determined using Statistical Package for the Social Sciences (SPSS, version 22.0, Armonk, NY, USA) by performing independent Student’s t-tests with a two-tailed distribution for the comparison of two mean values. A *p* < 0.05 was considered statistically significant.

## 5. Conclusions

We have developed a pharmacokinetic method for evaluating the pharmacokinetic characteristics of scutellarin and paeoniflorin, the main components of Xin-Shao formula extracts, in sham-operated and MCAO rats. Increased t_1/2z_ and MRT_(0–t)_ of scutellarin as well as elevated AUC_(0–t)_ and C_max_ of paeoniflorin were observed in cerebral I/R injury rats, relative to those in sham-operated animals, combined with decreased CL_z_ of both scutellarin and paeoniflorin as well as reduced V_z_ of paeoniflorin. The observed alternation in pharmacokinetics of the two bioactive components might result from decreased expression of P450 enzymes and UGTs under cerebral I/R injury conditions.

## Figures and Tables

**Table 1:** Precision and accuracy of UPLC-MS/MS method for assay the two analytes in rat plasma (mean ± SD, *n* = 5).

Components	Concentration Spiked (μg/mL)	Intra-Day			Inter-Day		
		Calculate Concentration (μg/mL)	Precision (RSD, %)	Accuracy (%)	Calculate Concentration (μg/mL)	Precision (RSD, %)	Accuracy (%)
Scutellarin	0.1160	0.1123 ± 0.0075	6.7	96.84	0.1093 ± 0.0097	8.9	94.25
	0.9277	0.9030 ± 0.037	4.1	97.34	0.94 ± 0.056	6.0	101.3
	14.84	14.10 ± 0.62	4.4	95.01	14.27 ± 0.91	6.4	96.14
Paeoniflorin	0.1180	0.1140 ± 0.0066	5.8	96.61	0.1127 ± 0.0064	5.7	95.48
	0.9440	0.9133 ± 0.040	4.4	96.75	0.888 ± 0.047	5.2	94.07
	15.11	15.33 ± 0.99	6.4	101.5	14.63 ± 1.1	7.6	96.85

SD: standard deviation, *n*: number of replicates, RSD: relative standard deviation.

**Table 2:** Extraction recoveries and matrix effect of the two analytes in rat plasma (mean ± SD, *n* = 5).

Components	Concentration Spiked (μg/mL)	Extraction Recovery (%)	Matrix Effect (%)
Scutellarin	0.1160	84.77 ± 6.6	88.79 ± 2.3
	0.9277	89.43 ± 2.4	91.23 ± 4.0
	14.84	90.52 ± 3.2	91.64 ± 3.4
Paeoniflorin	0.1180	86.72 ± 4.4	85.59 ± 2.5
	0.9440	89.95 ± 3.6	84.39 ± 2.9
	15.11	94.04 ± 1.6	87.05 ± 2.4

SD: standard deviation, *n*: number of replicates.

**Table 3:** Stability of scutellarin and paeoniflorin in rat plasma under various storage conditions (*n* = 5).

Components	Concentration Spiked (μg/mL)	6 h at 24 °C		Three Freeze-Thaw Cycles		Long-Term Stability (−20 °C, 2 weeks)	
		Precision (RSD, %)	Accuracy (%)	Precision (RSD, %)	Accuracy (%)	Precision (RSD, %)	Accuracy (%)
Scutellarin	0.1160	5.0	101.3	3.9	96.55	5.2	93.39
	0.9277	3.3	95.47	6.3	97.16	3.2	95.47
	14.84	4.3	98.54	5.4	96.59	3.7	96.91
Paeoniflorin	0.1180	5.1	100.3	7	103.1	6.5	94.07
	0.9440	4.6	95.94	4.4	97.35	5.3	99.93
	15.11	3.8	95.70	8.8	96.00	4.2	97.37

*n*: number of replicates, RSD: relative standard deviation.

**Table 4:** Pharmacokinetic parameters of scutellarin and paeoniflorin in rat plasma following intravenous administration of Xin-Shao formula extracts at a dose of 1.25 g/kg (mean ± SD, *n* = 6).

Parameters		Scutellarin		Paeoniflorin	
		Sham-Operated Group	MCAO Group	Sham-Operated Group	MCAO Group
AUC_(0–t)_	mg·min·L^−1^	3.88 ± 1.89	4.431 ± 0.716	10.35 ± 2.41	18.35 ± 3.71 **
MRT_(0–t)_	min	0.26 ± 0.09	0.42 ± 0.15 *	0.38 ± 0.12	0.35 ± 0.053
t_1/2z_	min	0.81 ± 0.48	1.66 ± 0.68 *	0.39 ± 0.0953	0.33 ± 0.091
CL_z_	L·min·kg^−1^	1.57 ± 0.60	0.97 ± 0.26 *	0.78 ± 0.16	0.45 ± 0.11 **
V_z_	L·kg^−1^	1.53 ± 0.66	2.90 ± 1.70	0.43 ± 0.07	0.21 ± 0.08 **
C_max_	mg·L^−1^	26.72 ± 12.18	32.34 ± 6.407	36.40 ± 5.71	56.28 ± 12.62 **

Values are mean ± standard deviation (SD, *n* = 6). *: *p* < 0.05, **: *p* < 0.01 compared with MCAO group. AUC_(0–t)_: area under the plasma drug concentration-time curve from 0 h to the terminal time point; MRT_(0–t)_: mean residence time from 0 h to the terminal time point; t_1/2z_: terminal elimination half-life; CL_z_: terminal clearance; V_z_: terminal volume of distribution; C_max_: maximum plasma drug concentration.

**Table 5:** Specific mass spectrometric parameters of selected ion recording (SIR) mode of the analytes and puerarin (IS).

Molecular	Polarity	Molecular Mass	Parent (*m*/*z*)	Dwell Time (s)	Cone Voltage (v)
Scutellarin	ESI+	462.04	463.04	0.2	22
Paeoniflorin	ESI−	526.12	525.12	0.1	26
Puerarin (IS)	ESI+	416.14	417.14	0.1	40

## Abbreviations

AUC_(0−t)_	area under the plasma drug concentration-time curve from 0 h to the terminal time point
C_max_	maximum plasma drug concentration
CL_z_	clearance
CCA	common carotid artery
CYP450	cytochrome P450
ESI	electrospray ionization
ECA	external carotid artery
I/R	ischemia and reperfusion
IS	internal standard
ICA	internal carotid artery
LLOQ	lower limit of quantification
MCAO	middle cerebral artery occlusion I/R injury
MRT_(0−t)_	mean residence time
*n*-BuOH	*n*-butanol
QC	quality control
RSD	relative standard deviation
SIR	selected ion recording
SD	standard deviation
TMC	traditional Chinese medicine
TTC	2,3,5-triphenyltetrazolium chloride
t_1/2z_	terminal elimination half-life
UPLC-MS/MS	ultra-performance liquid chromatography tandem mass spectrometry
UGTs	UDP-glucuronosyltransferases
V_z_	apparent volume of distribution

## References

[1] Donnan G.A., Fisher M., Macleod M., Davis S.M. (2008). Stroke. Lancet.

[2] Zeng M.F., Pan L.M., Zhu H.X., Zhang Q.C., Guo L.W. (2010). Comparative pharmacokinetics of baicalin in plasma after oral administration of Huang-Lian-Jie-Du-Tang or pure baicalin in MCAO and sham-operated rats. Fitoterapia.

[3] Fujimura M.K., Tominaga T.J., Chan P.H. (2005). Neuroprotective effect on antioxidant in ischemic brain injury: Involvement of neuronal apoptosis. Neurocrit. Care.

[4] Chen C., Venketasubramanian N., Gan R.N., Lambert C., Picard D., Chan B.P., Chan E., Bousser M.G., Xuemin S. (2009). Danqi Piantang Jiaonang (DJ), a traditional Chinese medicine, in poststroke recovery. Stroke.

[5] Wang Y.L., Huang Y., Zheng L., Wang A.M., Long Y.J., He X., Lan Y.Y. (2007). Study on the compound compatibility of Xinshao freeze-dried powder injection. Chin. J. Exp. Tradit. Med. Form..

[6] Wang H.J., Wang Y.L., Lan Y.Y., Wang A.M., Huang Y., Fang T.H., Xu L. (2008). Effect of lyophilized Xinshao injection on brain circulation in anesthetized dogs. Lishizhen Med. Mater. Med. Res..

[7] Wang H.J., Wang Y.L., Fang T.H., Xu L. (2008). Experimental research of effect of xinshao injection on nervous, respiratory and cardiovascular systems. J. Pharm. Res..

[8] Liu L.N., Lan Y.Y., Zheng L., Long Y.J., Huang Y. (2012). Interaction of compatibility of *Erigeron breviscapi* and radix paeoniae rubra on thrombus formation and coagulation time. Chin. J. Exp. Tradit. Med. Form..

[9] Huang Y., Wang Y.L., Lan Y.Y., Wang A.M., Fang T.H., Xu L. (2008). Effects of Xinshao injection on cerebral ischemia-reperfusion injury and regional cerebral blood flow in rats. Chin. J. New Drugs.

[10] Liu Z.Y., Dong L., Dong Y.X., Li L., Lan Y.Y., Wang A.M., Wang Y.L. (2014). Protective effects of the formula of herba erigerontis and radix paeoniae rubra on the injury induced by H_2_O_2_ of PC12 Cells. Chin. J. Exp. Tradit. Med. Form..

[11] Zheng L., Mu J.L., Tang Li., Liu Y., Wang A.M., Lan Y.Y. (2014). Simultaneous determination of two components of Xin Shao extract in rat plasma by UPLC-MS and their pharmacokinetics and absolute bioavailability. Chin. J. New Drug.

[12] Zhu H.X., Qian Z.L., He F., Liu W.Z., Pan L.M., Zhang Q.C., Tang Y.P. (2013). Novel pharmacokinetic studies of the Chinese formula Huang-Lian-Jie-Du-Tang in MCAO rats. Phytomedicine.

[13] He X., Xing D., Ding Y., Li Y., Du L. (2014). Effects of cerebral ischemia-reperfusion on pharmacokinetic fate of paeoniflorin after intravenous administration of paeoniae radix extract in rats. J. Ethnopharmacol..

[14] Tang H., Tang Y.P., Li N.G., Shi Q.P., Guo J.M., Shang E.X., Duan J.A. (2014). Neuroprotective effects of scutellarin and scutellarein on repeatedly cerebral ischemia-reperfusion in rats. Pharmacol. Biochem. Behav..

[15] Gan P., Zhong M., Huang X., Sun M., Wang Y., Xiao Y., Zeng C., Yuan Q., Liu Z., Zhou H. (2012). Pharmacokinetic comparisons of albiflorin and paeoniflorin after oral administration of Shaoyao-Gancao-Tang and single herb paeony decoction to rats. Planta. Med..

[16] Zheng L., Mu J.L., Huang Y., Dong L., He F., Lan Y.Y. (2014). Simultaneous determination of seven ingredients of Xin Shao freeze-dried power injection by UPLC-MS/MS. Chin. J. New Drug.

[17] Zhang Y.Q., Li H., Huang M.Q., Huang M., Chu K.D., Xu W., Zhang S.N., Que J.H., Chen L.D. (2015). Paeoniflorin, a monoterpene glycoside, protects the brain from cerebral ischemic injury via inhibition of apoptosis. Am. J. Chin. Med..

[18] Guo R.B., Wang G.F., Zhao A.P., Gu J., Sun X.L., Hu G. (2012). Paeoniflorin protects against ischemia-induced brain damages in rats via inhibiting MAPKs/NF-κB-mediated inflammatory responses. PLoS ONE.

[19] Yu J.B., Zhang Jun., Wang W.Q., Huang L.Q., Feng X.F. (2011). Comparative study on content of four constituent between wild and cutivated radix paeoniae rubra. Chin. J. Exp. Tradit. Med. Form..

[20] Liu J., Chen L., Fan C.R., Li H., Huang M.Q., Xiang Q., Xu W., Xu W., Chu K.D., Lin Y. (2015). Qualitative and quantitative analysis of major constituents of paeoniae radix alba and paeoniae radix rubra by HPLC-DAD-Q-TOF-MS/MS. Chin. J. Chin. Mater. Med..

[21] Niu W.M., Liu Z.B., Yang X.H., Wang Y., Shi H.L. (2013). Effect on CATmRNA expression in cerebral ischemia reperfusion rat with hepatic injury treated by protecting liver to nourish brain therapy. Liaoning J. Tradit. Chin. Med..

[22] Zhao W., Li F.J., Wang S. (2014). Protective effects of chrysophanol on liver injury induced by cerebral ischemia-reperfusion in mice. Acta Neuropharmacol..

[23] Zhong W.H., Qian K.J., Xiong J.B., Ma K., Wang A.Z., Zou Y. (2016). Curcumin alleviates lipopolysaccharide induced sepsis and liver failure by suppression of oxidative stress-related inflammation via PI3K/AKT and NF-κB related signaling. Biomed. Pharmacother..

[24] Gao C.Y., Chen X.Y., Zhong D.F. (2011). Absorption and disposition of scutellarin in rats: A pharmacokinetic explanation for the high exposure of its isomeric metabolite. Drug Metab. Dispos..

[25] Bock K.W. (2015). Roles of human UDP-glucuronosyltransferases in clearance and homeostasis of endogenous substrates, and functional implications. Biochem. Pharmacol..

[26] Gao C.Y., Zhang H.J., Guo Z.T., You T.G., Chen X.Y., Zhong D.F. (2012). Mechanistic studies on the absorption and disposition of scutellarin in humans: Selective OATP_2_B_1_-mediated Hepatic uptake is a likely key determinant for its unique pharmacokinetic characteristics. Drug Metab. Dispos..

[27] Richardson T.A., Sherman M., Kalman D., Morgan E.T. (2006). Expression of UDP-glucuronosyltransferase isoform mRNAs during inflammation and infection in mouse liver and kidney. Drug Metab. Dispos..

[28] Petrovic-Djergovic D., Goonewardena S.N., Pinsky D.J. (2016). Inflammatory disequilibrium in stroke. Circ. Res..

[29] Yang X.F., He W., Lu W.H., Zeng F.D. (2003). Effects of scutellarin on liver function after brain ischemia/reperfusion in rats. Acta Pharmacol. Sin..

[30] Gong W.H., Zheng W.X., Wang J., Chen S.H., Pang B., Hu X.M., Cao X.L. (2012). Coexistence of hyperlipidemia and acute cerebral ischemia/reperfusion induces severe liver damage in a rat model. World J. Gastroenterol..

[31] Bing Y.T., Zhu S.Y., Jiang K., Dong G.C., Li J., Yang Z.Q., Yang J., Yue J. (2014). Reduction of thyroid hormones triggers down-regulation of hepatic CYP2B through nuclear receptors CAR and TR in a rat model of acute stroke. Biochem. Pharmacol..

[32] Tan Y., Shen G.L., Zhuang X.M., Yuan M., Li H., Gao Y. (2013). Metabolic characteristics of paeoniflorin in vitro. J. Int. Pharm. Res..

[33] Lee J.H., Lee A., Oh J.H., Lee Y.J. (2012). Comparative pharmacokinetic study of paclitaxel and docetaxel instreptozotocin-induced diabetic rats. Biopharm. Drug Dispos..

[34] (1996). Guide for the Care and Use of Laboratory Animals.

[35] Longa E.Z., Weinstein P.R., Carlson S., Cummins R. (1989). Reversible middle cerebral artery occlusion without craniectomy in rats. Stroke.

[36] Joshi C.N., Jain S.K., Murthy P.S. (2004). An optimized triphenyltetrazolium chloride method for identification of cerebral infarcts. Brain Res. Protoc..

[37] Ding W.T., Zhou L.Q., Liu W., Guan L., Li X.Y., Liu H.M., Yan F.M., Xu J.W., Zeng W.Y., Qiu M. (2014). Opposite effects of the gap junction blocker octanol on focal cerebral ischemia occluded for different durations. Mol. Med. Rep..

